# Bronchiolitis obliterans associated with Stevens-Johnson Syndrome: histopathological bronchial reconstruction of the whole lung and immunohistochemical study

**DOI:** 10.1186/1746-1596-8-134

**Published:** 2013-08-06

**Authors:** Keishi Sugino, Akira Hebisawa, Toshimasa Uekusa, Kazuhito Hatanaka, Hiroshi Abe, Sakae Homma

**Affiliations:** 1 Department of Respiratory Medicine, Toho University Omori Medical Center, Omorinishi 6-11-1, Ota-ku, Tokyo 143-8541, Japan; 2 Department of Pathology, Tokyo National Hospital, Tokyo, Japan; 3 Department of Pathology, Labor Health and Welfare Organization Kanto Rosai Hospital, Kanagawa, Japan; 4 Department of Molecular and Cellular Pathology, Kagoshima Graduate School of Medicine and Dental Sciences, Kagoshima, Japan; 5 Department of Pathology, Juntendo University School of Medicine, Tokyo, Japan

**Keywords:** Stevens-Johonson syndrome, Bronchiolitis obliterans, Constrictive bronchiolitis obliterans, Bronchial reconstruction, Immunohistochemistry

## Abstract

This study presents an extremely rare case of constrictive bronchiolitis obliterans (BO) associated with Stevens-Johnson Syndrome (SJS) provides the morphological and immunohistochemical features using histopathological bronchial reconstruction technique. A 27-year-old female developed progressive dyspnea after SJS induced by taking amoxicillin at the age of 10. Finally, she died of exacerbation of type II respiratory failure after 17 years from clinically diagnosed as having BO. Macroscopic bronchial reconstruction of the whole lungs at autopsy showed the beginning of bronchial obliterations was in the 4th to 5th branches, numbering from each segmental bronchus. Once they were obliterated, the distal and proximal bronchi were dilated. Microscopic bronchial reconstruction demonstrated the localization of obliteration was mainly from small bronchi to membranous bronchioli with intermittent airway luminal narrowing or obliteration. Moreover, CD3-, CD20-, and CD68-positive cells were found in the BO lesions. CD34- and D2-40-positive cells were mainly distributed in the peribronchiolar lesions and bronchiolar lumens, respectively. SMA- and TGF-β-positive cells were seen in the fibrous tissue of BO lesions.

**The virtual slides:**

The virtual slide(s) for this article can be found here: http://www.diagnosticpathology.diagnomx.eu/vs/1071703140102601.

## Background

Stevens-Johnson syndrome is also called mucocutaneous ocular syndrome and causes severe erythema exsudativum multiform [1]. SJS is caused by various drugs including antimicrobial or antiepileptic drugs and infectious diseases such as mycoplasma and viruses [2]. Although pulmonary complications are often observed in SJS, bronchiolitis obliterans (BO) is extremely rare and its incidence is not still understood.

We described a patient of constrictive BO associated with SJS that progressively deteriorated during long-term period and demonstrated characteristic histopathological features by bronchial reconstruction and immunohistochemical stain.

## Case presentation

**Figure 1:**
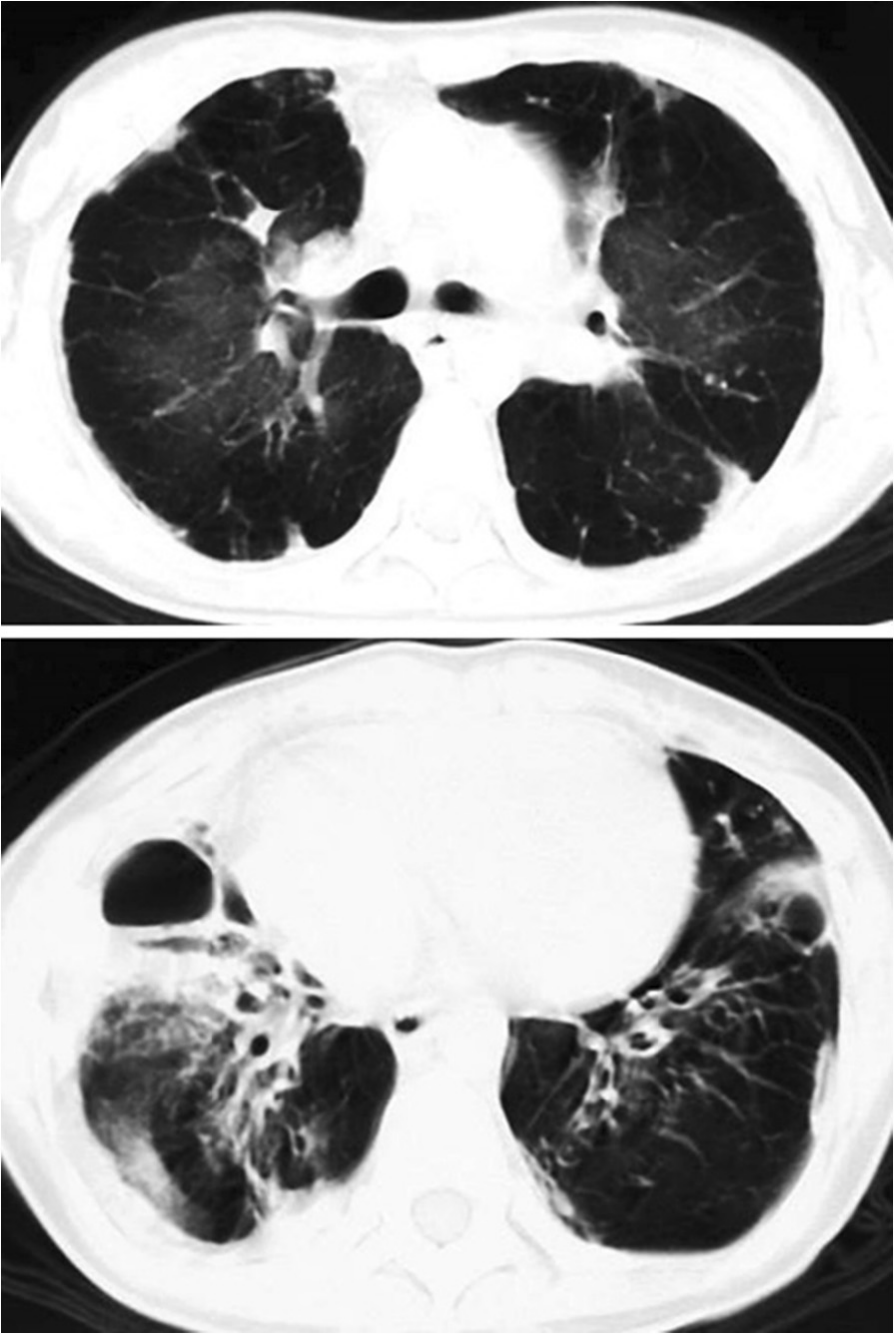
Chest CT scan revealed a widespread mosaic pattern with air trapping and diffuse pleural thickening in both lungs and prominent broncho-bronchiolectasis in the bilateral lower lobes predominance.

A 25-year-old female had a history of SJS after oral administration of amoxicillin at the age of 10. Two months after the onset of SJS, she began to suffer from dyspnea on exertion and bilateral pneumothorax repeatedly. The patient was referred to our hospital due to fever and progressive dyspnea at the age of 25. On admission, the pulmonary function tests showed mixed ventilatory and small airways impairment as follows: vital capacity (VC) of 1.12 L (36.8% of predicted), forced expiratory volume in 1 second (FEV_1_) of 0.60 L (53.1% of predicted), residual volume/total lung capacity (RV/TLC) of 52.3%.Blood gas analysis showed PaO_2_ of 79.5 Torr and PaCO_2_ of 60.1 Torr under inhaling oxygen on 1.5 L/min. Chest X-ray revealed severe hyperinflation in both lung fields and pleural adhesion in the right lower lung field. Chest computed tomography (CT) scan revealed a widespread mosaic pattern with air trapping and diffuse pleural thickening in both lungs and prominent broncho-bronchiolectasis in the bilateral lower lobes predominance (Figure 1). During the clinical course, she underwent thoracoscopic cyst stitch surgery for right pneumothorax. Afterward, a single dose of carbapenem antibiotics administered due to postoperative infection triggered an anaphylactic shock. She was immediately treated with corticosteroids and her symptoms improved. However, withdrawal from a mechanical ventilator was difficult due to postoperative pneumonia, she underwent tracheotomy with continuous mechanical ventilation. At the same time, the patient was clinically diagnosed as having BO associated with SJS. Although azithromycin was administered to treat a chronic respiratory tract infection, *Pseudomonas aeruginosa* were frequently positive in sputum cultures. After 2 years from the initial admission to our hospital, she suffered septic shock with an exacerbation of type II chronic respiratory failure. Finally, she died after 17 years from the onset of BO.

### Histopathology and immunohistochemistry

The autopsied lungs were fixed in 10% formalin solution and stained with hematoxylin and eosin (H&E) and elastic van Gieson (EVG). After macroscopic examination with bronchial reconstruction, 2 cm serial sections along each bronchus were obtained from each lobe in both lungs for the microscopic reconstruction studies. Tissue samples for microscopic analyses were embedded in paraffin, and sliced into 1,200 serial sections with a thickness of 4 μm as described previously [3]. Morphological analysis was performed under a conventional light microscope to determine the characteristics of the BO lesions, including degrees of bronchiolar epithelial cell damage, fibrosis, inflammation in the lumen and wall, destruction of wall structure, and dilated airway. The localization and distribution of the BO lesions were histologically reconstructed. A membranous bronchiole (i.e. non-respiratory bronchiole) was defined as an airway with a diameter of 2 mm or less and lack of both cartilage and bronchial glands, and a small bronchus was defined as a cartilaginous airway with diameter of 2 mm or more, which was located in more proximal of membranous bronchiole.

4 μm-thick paraffin sections were immunohistochemically stained with the following mouse monoclonal antibodies: T cells with anti-CD3 (clone UCH-T1, Santa Cruz Biotechnology), anti-CD4 (clone MT310, Santa Cruz Biotechnology), anti-CD8 (clone 5F10, Santa Cruz Biotechnology); B cell with anti-CD20 (clone L26, Dako); macrophages/histiocytes with anti-CD68 (clone KP-1, Dako); myofibroblasts with α-smooth muscle actin (SMA) (clone 1A4, Dako); vascular and lymphatic endothelial cells, anti-CD34 (clone NU-4A1, Nichirei), and anti-D2-40 (clone D2-40, Dako), and transforming growth factor-beta 1 (TGF-β1) (clone 9016, R&D). Immunohistochemicalstaining was graded on a semi-quantitative scale of as follows: absent, weak (weak staining in < 50% of cells), moderate (weak staining in > 50% or strong staining in < 50% of cells) and intense (strong staining in > 50% of cells). Randomly selected high power fields were evaluated.

### Pathological findings

#### Macroscopic bronchial reconstruction

**Figure 2:**
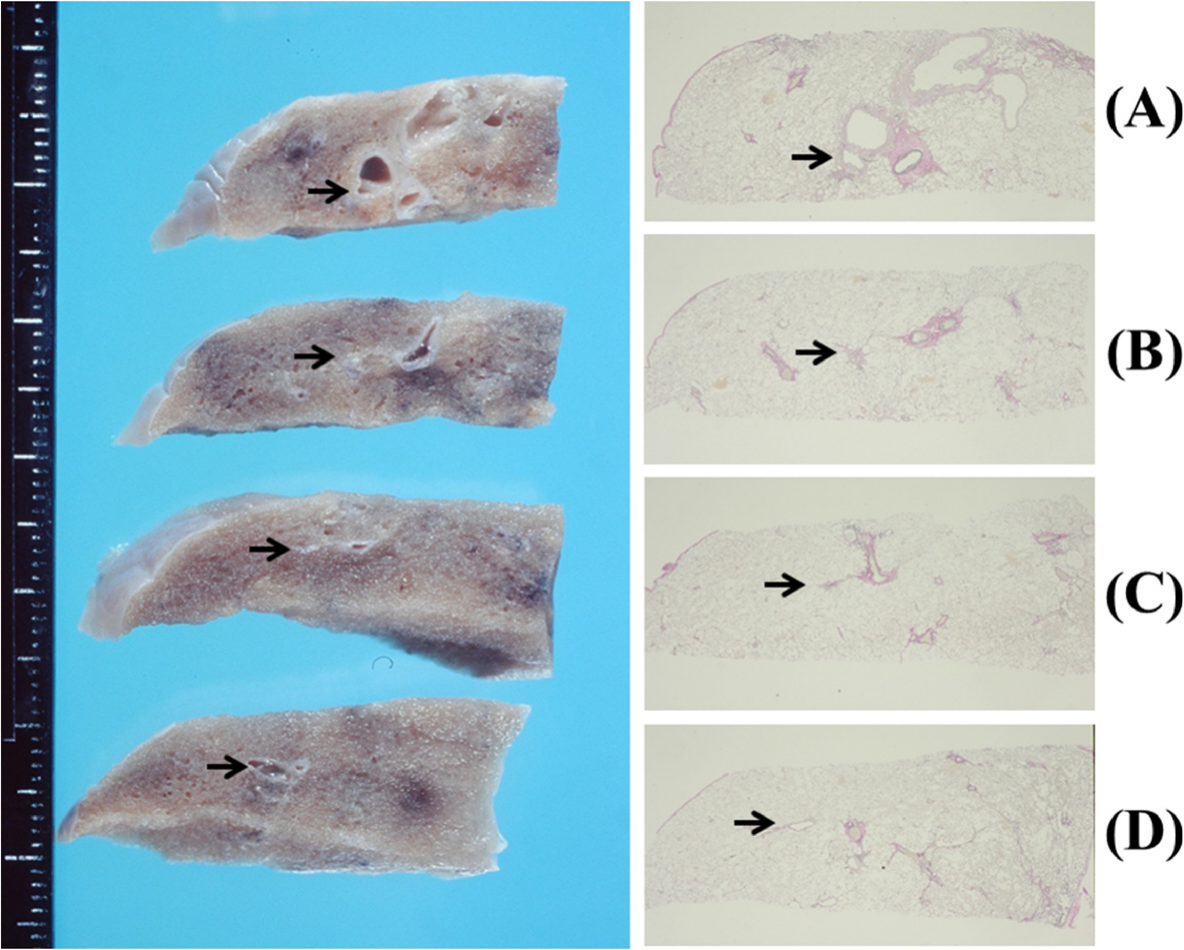
**Comparison between macroscopic and full-scale microscopic appearance of the left superior horizontal bronchiole. (A)** A small bronchus was dilated and the lumen surrounded by fibrosis. **(B)** The localization of the BO lesions with airway luminal narrowing was mainly observed in a membranous bronchiole corresponding to macroscopic appearance of a whitish small nodule. **(C)** The membranous bronchiolar lumen was completely obliterated. **(D)** The more distal bronchiole than the membranous bronchiole was dilated. (arrows) (Scale bar = 1 mm) (Elastic van Gieson stain).

Grossly, autopsied lungs showed diffuse pleural adhesions as well as multiple, and whitish nodules in both lungs. Most of the bronchi and bronchioli in the right middle lobe and both lower lobes showed tubular dilatation and partially cyst-like dilatation with thickened walls. No other apparent abnormalities such as pneumonia were seen in both lungs. Macroscopic bronchial reconstruction showed the beginning of bronchial obliterations was in the 4th to 5th branches when each segmental bronchi are regarded as the first branch. As compared with macroscopic and full-scale microscopic appearance of the left superior horizontal bronchiole, the localization of the BO lesions with airway luminal narrowing were mainly observed in membranous and more proximal bronchioli corresponding to macroscopic appearance of a whitish small nodule. After the membranous bronchiolar lumen was completely obliterated, again the more distal bronchiole than the membranous bronchiole was dilated. As a result, the occlusion of the bronchiole was located discretely (Figure 2). No other abnormalities were seen in the systemic organs other than both lungs.

#### Microscopic bronchial reconstruction

**Figure 3:**
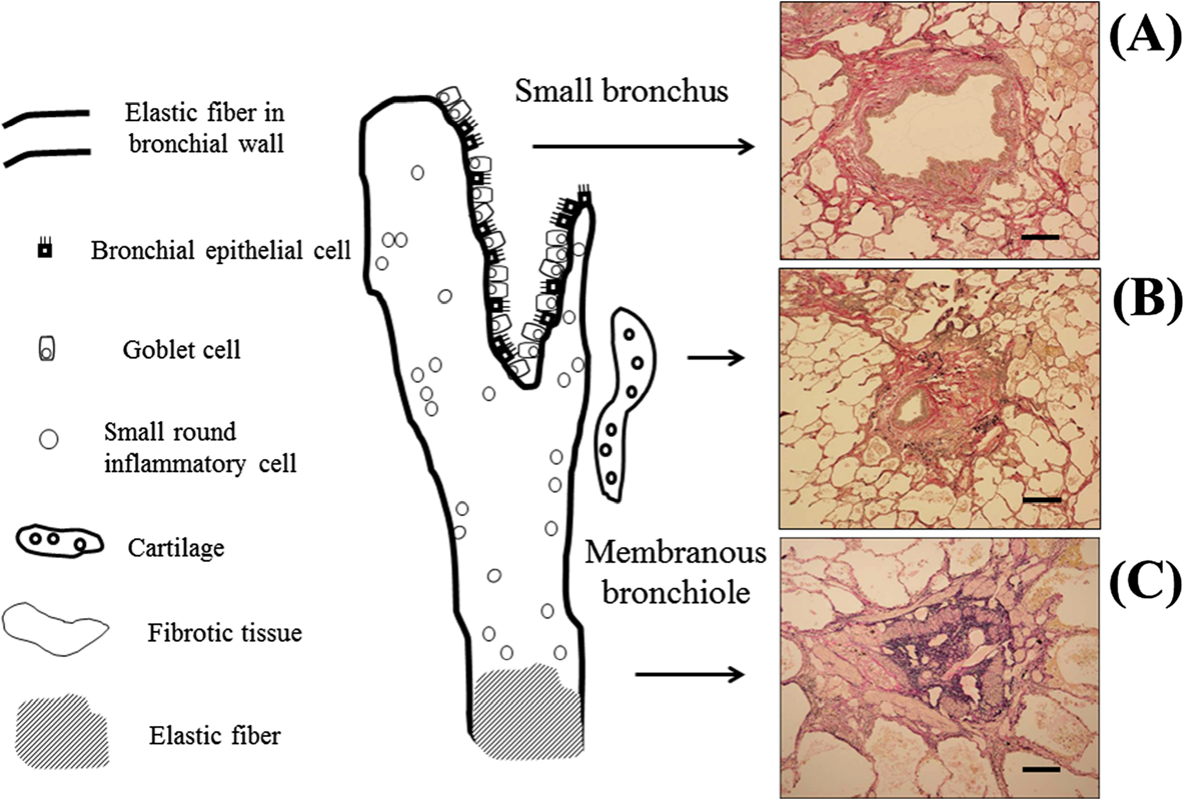
**Microscopic bronchial reconstruction. (A)** Small bronchi were dilated with mild infiltration of small round inflammatory cells and their epitheliums were replaced by goblet cell hyperplasia. (Scale bar = 500 μm) (Elastic van Gieson stain). **(B)** The prominent concentric fibrosis with mild infiltration of small round inflammatory cells was present in the submucosal layers, resulting in membranous bronchiolar luminal narrowing and complete occlusion. (Scale bar = 500 μm) (Elastic van Gieson stain). **(C)** The marked proliferation of elastic fibers, lack of inflammatory cells infiltration was observed in the more distal than membranous bronchioli. (Scale bar = 200 μm) (Elastic van Gieson stain).

The airway lumens of the BO lesions were obliterated by the fibrous tissues accompanying a proliferation of the elastic fibers with mild infiltration of small round inflammatory cells and accumulation of foamy macrophages, and the structures of the bronchiolar wall were preserved, indicating constrictive BO. On the other hand, the bronchial wall structure was completely destroyed and replaced by fibrous granulation tissue accompanied by infiltration of foamy macrophages, lymphocytes, and plasma cells with intraluminal neutrophils as well as collections of mucus in the dilatation of bronchi of the right middle lobe and both lower lobes. Constrictive BO was observed at the periphery of the bronchiolectasis. Moreover, a colony of *Actinomyces* was found in the right lower lobe. On microscopic bronchial reconstruction, small bronchi were dilated with mild infiltration of small round inflammatory cells and their epitheliums were replaced by goblet cell hyperplasia. The prominent concentric fibrosis with mild infiltration of small round inflammatory cells was present in the submucosal layers, resulting in membranous bronchiolar luminal narrowing and complete occlusion. In addition, the marked proliferation of elastic fibers with a few inflammatory cells infiltration was observed in the more distal bronchioli than membranous bronchioli. Some BO lesions were sporadically and intermittently located with varying degrees of narrowing and/or obliteration from the small bronchi to the membranous bronchioli. There was no evidence of BO lesions in respiratory bronchioli and alveoli (Figure 3).

#### Comparison between chest CT images and histopathological findings

A widespread mosaic pattern with air trapping and diffuse pleural thickening in both lungs on chest CT corresponded to obliterative bronchiole and diffuse pleural adhesions. In addition, varicose or cylindrical bronchiectasis in the bilateral lower lobes on chest CT consisted of bronchiolectasis with tubular and cystic dilatation.

#### Immunohistochemical studies

CD3, CD4, CD8, CD20 and CD68 were localized in lymphocytes and macrophages infiltrating the BO lesions of membranous bronchioli.

**Figure 4:**
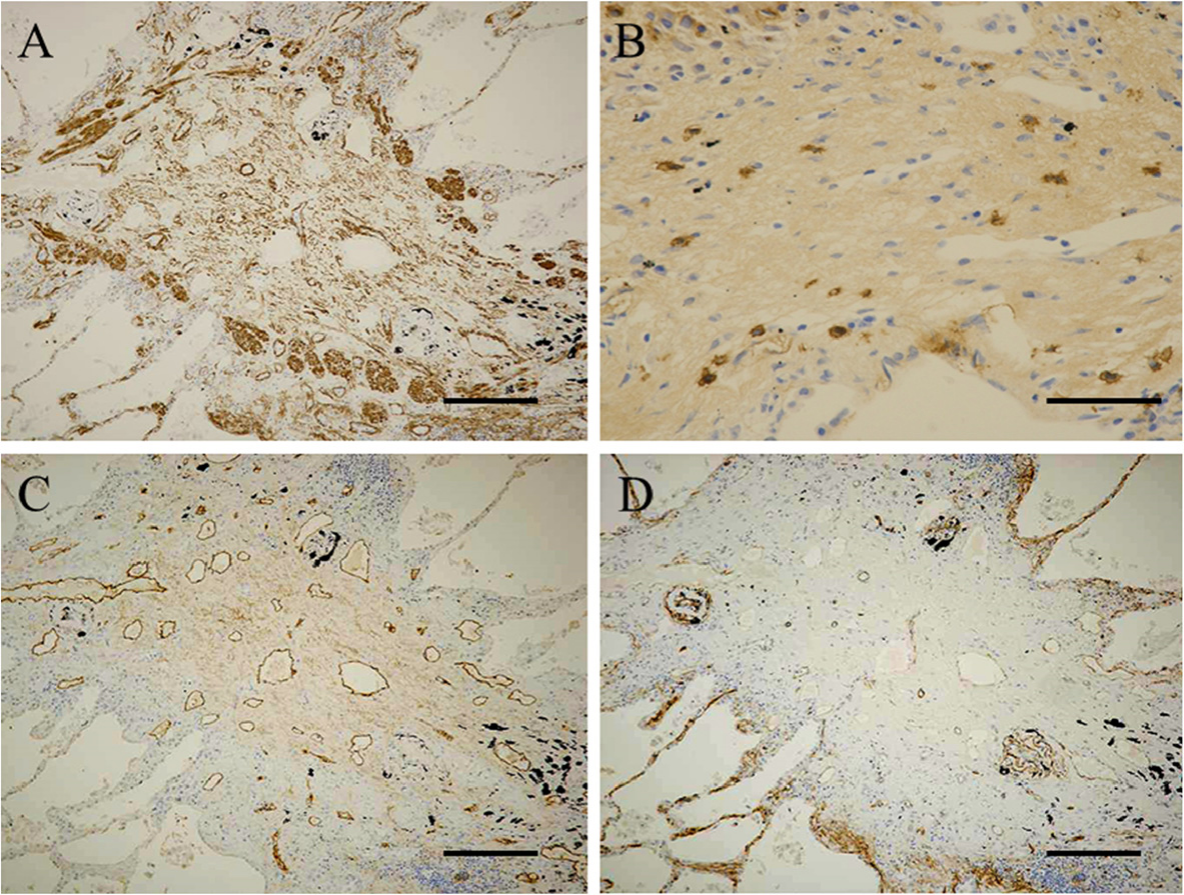
**Immunohistochemical findings. (A)** Numerous SMA-positive myofibroblasts (intense) were present in airway lumens (Scale bar = 100 μm). **(B)** TGF-β-positive lymphocytes and macrophages (moderate) were sporadically seen in the fibrous tissue of BO lesions (Scale bar = 20 μm). **(C)** CD34-positive cells (moderate) were mainly distributed in the peribronchiolar lesions (Scale bar = 100 μm). **(D)** D2-40-positive cells (intense) were uniformly distributed in the bronchiolar lumens and peribronchiolar lesions (Scale bar = 100 μm).

The expression of CD8 (moderate) was higher than that of CD4 (mild) in the BO lesions. Numerous SMA-positive myofibroblasts (intense) were present in airway lumens (Figure 4A) and TGF-β-positive lymphocytes and macrophages (moderate) were sporadically seen in the fibrous tissue of BO lesions (Figure 4B). CD34-positive cells were mainly distributed in the peribronchiolar lesions, in contrast, D2-40-positive cells were uniformly distributed in the bronchiolar lumens and peribronchiolar lesions. The expression of D2-40 (intense) was higher than that of CD34 (moderate) in the BO lesions (Figure 4C, D).

## Discussion

SJS is known as mucocutaneous ocular syndrome and causes severe erythema exsudativum multiform [1]. The syndrome is caused by many drugs, including antibiotics or antiepileptic, as well as by infection such as viruses or mycoplasma [2]. The pathogenesis of chronic pulmonary disorders such as BO in SJS remains obscure. BO is a rare disease in which granulation tissue mainly obliterates the lumen of the membranous bronchioli. It is useful to diagnose as having BO clinically based on mosaic appearance on chest CT images or small airway obstructive impairment on pulmonary function test, but as confirmed diagnosis of BO requires lung specimens obtained by video-assisted thoracic surgery or autopsy, it may be difficult to make a correct antemortem diagnosis. Theegarten et al. [4] reported that diagnosis of interstitial pneumonias, a group of quite rare diseases, by open lung biopsies required sufficient clinical information because of the overlap of histological patterns and especially in non idiopathic interstitial pneumonias, an interdisciplinary case evaluation was needed. Therefore, we believe it is necessary to find a correct diagnosis histologically. More recently, Griff et al. [5] reported that specimens obtained by transbronchial cryobiopsy technique were significantly larger than those by conventional transbronchial biopsies, in addition the alveolar tissue of cryobiopsy specimens did not show any artifacts. This technique may be useful for the diagnosis of BO.

Yamanaka, et al. [6] conducted a histopathological study of BO associated with rheumatoid arithritis or SJS, and proposed bronchobronchiolitis obliterans (BBO) as the pathological condition in which the BO lesions were seen from the small bronchi to membranous bronchioli. A small number of BO cases caused by drug-induced SJS which were histologically confirmed have been reported so far [7-9]. As reported by Tsunoda, et al. [7], autopsied lung in BO associated with SJS showed macroscopically airway obliterations were located in the 3rd and 5th branches, numbering from each lobar bronchus. Histological examination revealed loss of bronchial epithelium and narrowing or obliteration of the bronchiolar lumen due to proliferation of granulation tissue not only in membranous bronchioli but also cartilaginous bronchi. In the present case, macroscopic appearances showed extensive occlusion of the bronchi at the 4th or 5th branches, which were located in more distal bronchi than each segmental bronchus. Histologically, the bronchial wall structure such as the smooth muscle layer and the elastic fiber layer was maintained, and the airway lumen was markedly occluded due to fibrous tissue, suggesting constrictive BBO. Interestingly, the BO lesions were sporadically and intermittently located from the small bronchi to the membranous bronchioli. On the other hand, bronchiectasis and the bronchial wall structures were completely destroyed and replaced by fibrous tissue in the right middle lobe and in both lower lobes in our case. We speculated that after SJS caused constrictive BBO throughout most of the lungs, bacterial infection such as *Pseudomonas aeruginosa*, etc. induced inflammations in the airways repeatedly, resulting in bronchial mucosal ulcers, bronchiectasis and bronchiolectasis during the following 17 years. Hebisawa, et al. [10] histopathologically searched for patients who died of diffuse bronchiectasis associated with chronic respiratory infections, and found destructive BO showing complete destruction of the bronchiolar wall structures and its lumen replacement with collagen fibers.

The pathogenic mechanism of BO in SJS is not well understood, but we believe that BO in SJS appears as a consequence of bronchiolar epithelium and mucosal damage due to immune complex deposition in SJS. Furthermore, the combination of the immune response abnormality and respiratory infection may play an important role in its development. According to previous reported cases, respiratory symptoms appeared within a few weeks to months after mucosal or skin symptoms improved. Also in the present case, respiratory symptoms appeared 2 months after treatment with large amounts of corticosteroid for SJS. We assumed that some kind of injuries to bronchiolar epithelial cells initiate its changes in necrosis and shedding of them, and then followed exudation of the fibrin and inflammatory cells such as lymphocytes, macrophages, and proliferation of the myofibroblasts and capillary vessels. Finally, the bronchiolar lumens were obliterated by fibrous granulation tissue. Therefore, it is considered that formation of BO requires certain intervals after the onset of SJS. However, the mechanisms of selective damages to mainly membranous bronchioli in BO remain obscure. Recently, Nicod [11] described that using heterotopic tracheal transplant models with mice obliteration of airway lumen may result from the overactivation of repair mechanisms by fibroblasts in damaged bronchioli and progressive structural reconstruction (remodeling) or fibrosis around the lesions. On immunohistochemical study of the present case, CD3-, CD20-, CD68-, and TGF-β positive cells were found partly but prominent SMA-positive cells infiltration in the BO lesions. In fact, the differences in the disease duration of BO or the immunocompromised state of the host associated with treatments or underlying disorders will influence the extent of inflammatory cells and myofibroblasts. Finally, production of growth factors such as TGF-β which promote fibroblast proliferation may result in fibrosis at late phase of BO formation. In addition, Shah et al. [8] reported that a case of constrictive BO and eosinophilic micro-abscesses after SJS. It is known that eosinophils are important sources of a variety of pro-fibrogenic mediators such as TGF-α [12], TGF-β [13], vascular endothelial growth factor [14], and interleukin-13 [15]. Although eosinophils were not found in the BO lesions at autopsy in the present case, increased eosinophils could be determined at an earlier stage of BO.

## Conclusion

This study provides important information on the morphological and immunohistochemical features in an extremely rare constrictive BO associated with SJS using histopathological bronchial reconstruction technique. The involvement of epithelial-mesenchymal transition or pro-fibrotic growth factors will need to be investigated in the future to clarify the mechanism of development of fibrotic lesions in BO associated with SJS.

### Consent

Written informed consent was obtained from the patient's next-of-kin for publication of this manuscript and any accompanying images. A copy of the written consent is available for review by the Editor-in-Chief of this journal.

## Abbreviations

SJS: Stevens-Johnson syndrome; BO: Bronchiolitis obliterans; BBO: Bronchobronchiolitis obliterans; CT: Computed tomography; H&E: Hematoxylin and eosin; EVG: Elastic van Gieson; TGF-β: Transforming growth factor-beta; SMA: α-smooth muscle actin.

## Competing interests

All authors declare that they have no competing interest.

## Authors’ contributions

K S participated in the design of the study and histopathological evaluation, and drafted the manuscript. S H assisted in drafting the manuscript and revised the manuscript. A H, T U, and K H made contributions for analyzing the histopathological characteristics. H A sliced each lung specimen into serial sections and carried out the H&E, EVG, and immunohistochemical stains evaluation. All the authors read and approved the final manuscript.
